# Couple Relationships Are Associated With Increased REM Sleep—A Proof-of-Concept Analysis of a Large Dataset Using Ambulatory Polysomnography

**DOI:** 10.3389/fpsyt.2021.641102

**Published:** 2021-05-10

**Authors:** Henning Johannes Drews, Annika Drews

**Affiliations:** ^1^Department of Mental Health, Norwegian University of Science and Technology (NTNU), Trondheim, Norway; ^2^Department of Psychiatry and Psychotherapy, Christian-Albrechts-University, Kiel, Germany; ^3^Department of Climate and Environment, SINTEF Ocean AS, Trondheim, Norway

**Keywords:** bed-sharing, REM sleep, couple relationship, sleep heart health study, marriage, co-sleeping

## Abstract

**Background/Objectives:** Rapid Eye Movement (REM) sleep is associated with memory consolidation and several health effects including stress response, mental health, and longevity. Recently, it has been shown that regularly co-sleeping couples have increased and stabilized REM sleep when co-sleeping as compared to sleeping individually. However, it remained unclear whether this is due to a specific effect of altering the usual sleeping environment by partner deprivation or due to a generalizable REM-sleep promoting effect of couple relationships. The present study aims to clarify this ambiguity.

**Methods:** Married or never married individuals were taken from the Sleep Heart Health Study (*n* = 5,804) and matched regarding sociodemographic and health parameters. Matching was done using propensity score matching (1:1, nearest neighbor) and resulted in two groups of *n* = 69 each (married vs. never married). After confirmation of successful matching, samples were compared regarding REM sleep and other polysomnographic parameters (paired Students *t*-tests or Wilcoxon signed-rank tests).

**Results:** Married individuals showed significantly higher levels of total and relative REM sleep as compared to never married individuals (all *p*'s ≤ 0.003). Neither other sleep stages nor REM-sleep fragmentation differed between groups (all *p*'s ≥ 0.29). Results regarding number of sleep cycles were ambiguous.

**Conclusion:** This is the first between-subjects study to show that couple relationships are associated with increased REM sleep. This finding represents a necessary (but not sufficient) condition for the previously hypothesized self-enhancing feedback loop of REM sleep and sociality as well as for REM-sleep promotion as a mechanism through which couple relationships prevent mental illness.

## Introduction

Sleep research has found REM sleep to be a key factor in central cognitive and health-related functions such as memory consolidation (1), development of major public health challenges as insomnia or depression (2–5), stress response (6), and even longevity (7). Particularly its close relationship to mental disorders make REM sleep a focus of interest in psychiatry (2, 3, 5, 8–10).

At the same time, REM sleep is a fragile state that is easily disturbed amongst others by psychosocial stress and therefore depends strongly on situational and environmental factors (6, 11, 12).

Despite the fact that about 50% of the adult population in western countries live in couple relationships and share a bed, the social sleep environment has only been marginally considered in this context. Particularly polysomnographic data are rare. A recent publication finds that regularly co-sleeping couples show increased and stabilized REM sleep when sleeping together as compared to sleeping apart (13). This reproduced findings from more than 50 years ago (14). Yet, both study designs make it impossible to differentiate whether the observed effects are due to psychosocial stress induced by changing the habitual sleeping environment (i.e., by partner deprivation) or whether they are due to a general promoting effect of close social relationships on (REM) sleep. The latter could have far reaching implications and could represent an important mechanism through which partnerships impact sociability and (mental) health (13). In fact, it has long been argued that sleep might mediate the health effects of couple relationships (15), yet mechanisms on the sleep stage level have remained a long-standing question.

Against this background, the present work seeks to clarify whether there is a general effect of couple relationships on (REM) sleep. The main hypothesis is: stable couple relationships are associated with higher levels of REM sleep as compared to not being in such a relationship.

The secondary hypothesis is that there are no additional changes in other sleep stages. Additionally, we investigate parameters that coincided with the increased amount of REM sleep during co-sleep in one of the previous studies: REM-sleep fragmentation (13) and number of REM-sleep periods (i.e., number of ultradian sleep cycles (14). Investigation of individual sleep cycles seems also of interest since it has been shown that—in people with mental illness—deficits in sleep stability are particularly pronounced in the first and third sleep cycle (16).

In order to test these hypotheses, we re-analyze a large dataset using in home polysomnography and compare never married to married individuals. That way, the habitual sleeping arrangement remains unaltered and both marital statuses are associated with ~95 and 90% of individually sleeping and bed-sharing, respectively (17, 18).

## Methods

### Procedure

The aim of the present study is to investigate the effect of being in a stable couple relationship on REM sleep. In order to do so, access to a large-sample observational study that used in-home polysomnography and collected marital status [the Sleep Heart Health Study; SHHS (19, 20)] was obtained through the National Sleep Research Resource (21, 22). Prior to filing for access to the data, the institutional review board (IRB) of the medical faculty of Kiel University confirmed that in light of the previous IRB clearance of the original trial and the anonymous nature of the dataset, no formal IRB clearance was needed for the present analysis. This is in accordance with European law.

### The Original Study

The dataset of the present study was derived from the baseline examinations of the Sleep Heart Health Study [SHHS; (19, 20)]. The SHHS was designed to longitudinally investigate obstructive sleep apnea as a risk factor for cardiovascular disease. Inclusion criteria were ≥40 years of age. Patients with diagnosed and treated sleep apnea were excluded but snorers below the age of 65 (19, 20) were purposively overrepresented in the study population.

Data acquisition of the SHHS took place between December 1995 and January 1998. During that time 6697 participants underwent overnight in-home polysomnography that was administered by a trained technician. Ninety five percent of the polysomnographies had an acceptable data quality and were included in the study. For further information please see Redline and colleagues (20) and Quan and colleagues (19).

### Measures Used for the Present Analysis

#### Polysomnography

Polysomnographic monitoring of the SHHS comprised EEG (C3/A1 and C4/A2), binocular electrooculography (EOGs), submental electromyography (EMG), monitoring of movements of chest and abdomen (via inductive plethysmography bands), airflow, pulse oximetry, ECG, and body position (20). Scoring was done manually by trained raters according to Rechtschaffen and Kales criteria (23). Sleep stages S3 and S4 were summed up [in the following referred to as slow-wave sleep (SWS)]. There was an “excellent” (24) intra- and interrater reliability regarding the scoring of sleep stages (kappa statistics >0.80) and respiratory events (intra class correlation >0.90). Arousal scoring was less reliable (intra class correlation = 0.54) (24).

Parameters that were used for further analysis in the present study were sleep-onset latency (min), total sleep time (min), sleep efficiency (%), sleep stages [S1, S2, SWS, and REM sleep; as total duration (min) and relative duration of total sleep time (%)], REM-sleep latency (min), wake after sleep onset (WASO, min), awakenings per hour of sleep (n), and Apnea-Hypopnea Index (AHI, n/h). We did not use arousals due to the limited reliability of the scoring. Additionally, we calculated the following parameters based on the manually scored sleep profiles of the SHHS: number of REM-sleep periods (i.e., number of sleep cycles), duration of sleep cycles, and REM-sleep fragmentation (i.e., total number of disruptions of REM sleep, relative number of disruptions per minute of REM sleep, and REM-sleep fragmentations per REM period). REM-sleep fragmentation was defined as intrusion of non-REM sleep or wake epochs within a REM-sleep period. This was in line with the previous operationalization of Drews and colleagues (13). A sleep cycle was defined by a sequence of non-REM sleep stages and the following REM sleep period (25).

#### Non-PSG Measures

The following sociodemographic parameters were used for the present study: gender [male (1), female (2)], marital status [married (1), never married (2)], ethnicity [white (1), non-white (2)], educational level [<10 years of education (1), 11–15y (2), 16–20y (3), >20y (4)], and age (years). For a comprehensive assessment of subjective health status the SF-36 (26) was used. The SF-36 is a widely-used instrument that measures eight dimensions of health, i.e., physical functioning, bodily pain, role limitations due to physical health problems, role limitations due to personal or emotional problems, general mental health, social functioning, energy/fatigue, and general health perceptions. Scores for each sub-scale range from 0 to 100. Higher scores represent better health status. In addition to using the results of the individual scores, we also used the sum score of all individual scores.

Additional health and subjective sleep parameters we used were body mass index (BMI; kg/m^2^), subjective daytime sleepiness [measured by the Epworth Sleepiness Scale (27)], as well as use of antidepressants and benzodiazepines [due to their (potential) effect on REM sleep (28, 29)].

### Sample Construction for the Present Study

Of the baseline SHHS participants, only those that showed no or mild respiratory symptoms (AHI <15 /h), that had no missing values regarding the used parameters, and that were either currently married (*n* = 1,889) or never married (*n* = 69) were used for the present study. To control for health and sociodemographic parameters, nearest neighbor propensity score matching (constantly married vs. never married; 1:1) was employed.

Propensity score matching is a statistical technique to retrospectively balance characteristics of groups that have not originally been balanced [for overviews see (30, 31)]. Propensity score matching is often employed to assess effects of a particular treatment in observational studies. Thus, it is a method to retrospectively create more randomized-controlled-trial (RCT) -like conditions in observational studies (30). It is particularly useful when trying to control for a large number of covariates between groups and it has been named a good alternative in absence of randomization (32).

Technically, propensity score matching consists of two steps. First, a logistic regression is used to define typical characteristics of a group with a certain target characteristic (e.g., having received a certain treatment, or—as in the present case—having never been married). Based on the results of the logistic regression, a score is calculated for each individual that describes the propensity of having the target characteristic. The second step is the matching procedure. In the present case, we used nearest neighbor matching with a 1:1 ratio. That means that to each participant that is positive with respect to the target characteristic, exactly one negative counterpart is allocated that has a similar (or very close) propensity score. It is of note that one of the most important factors that define quality of the matching procedure is the existence of a sufficiently large pool of controls from which the matching counterparts can be extracted. A factor of larger than 3–4:1 (control pool:treated group) has been suggested (31). In the present study, that factor is larger than 25:1 (1889:69; married:never married).

Initial matching parameters were: age, gender, education level, ethnicity, the SF-36 sum score, the SF-36 general mental health subscore, the SF36 general health subscore, and AHI.

After initial matching, imbalances between the groups regarding any of the other above-mentioned (non-PSG) parameters were checked. Detailed sample characteristics are given in Table 1.

**Table 1:** Sociodemographic and health parameters of the matched sample.

	**Total sample (*n* = 138, *m* = 48)**	**Constantly married (*n* = 69, *m* = 25)**	**Never married (*n* = 69, *m* = 23)**	***p*-value (married vs. unmarried)**
	**[median (Q1–Q3)]**	**[median (Q1–Q3)]**	**[median (Q1–Q3)]**	
Age	58.0 (47–57.9)	59 (50–64)	52 (44–72)	0.308
Education level	3 (2–3)	3 (2–3)	3 (2–3)	0.692
Body-Mass Index	25.7 (23.2–29)	25.6 (23.8–28.32)	25.7 (23.2–29.8)	0.83
Epworth Sleepiness Scale	6 (4–9)	6.5 (5–9.75)	6 (4–9)	0.267
Apnea-Hypopnea Index	6.9 (3.4–11.3)	6.6 (4–11)	7.2 (3–11.4)	0.877
SF 36 Sum	654.2 (562.9–717.8)	669 (562.7–718.0)	651 (568.3–706.5)	0.711
SF 36-Pain	80 (61–100)	80 (61–100)	84 (61–100)	0.805
SF 36-Physical functioning	90 (75–98.75)	90 (65–95)	90 (75–100)	0.491
SF 36-Role limitations due to physical health	100 (50–100)	100 (75–100)	100 (50–100)	0.371
SF 36-Role limitations due to emotional problems	100 (75–100)	100 (66.67–100)	100 (100–100)	0.505
SF 36-Energy/fatigue	65 (55–75)	65 (55–75)	70 (50–80)	1
SF 36-Emotional well-being	84 (72–88)	84 (72–88)	84 (72–88)	0.909
SF 36-Social functioning	100 (78.1–100)	100 (87.5–100)	100 (75–100)	0.430
SF 36-General health	77 (67–87)	77 (67–90)	80 (67–87)	0.513

*Education level is expressed on a four-point scale: 1 = <10 years of education, 2 = 11–15 y, 3 = 16–20 y, 4 = >20 y. Tests: Wilcoxon signed-rank test. Comparison of binary variables (gender and ethnicity—white vs. non-white (white: n = 56 in married couples; n = 54 in never married individuals), use of tricyclic antidepressants (n = 4; n = 2), non-tricyclic antidepressants (n = 1; n = 3), or benzodiazepines (n = 4; n = 6) by Chi-Squared or Fisher's tests showed no significant differences (all p's ≥ 0.62)*.

### Statistical Analyses

Normal distribution was tested using Shapiro-Wilk tests. Groups were compared using paired Student's *t*-tests, or—where applicable—the non-parametric alternative Wilcoxon signed-rank test. The primary hypothesis was tested using one-sided tests. The one-sidedness was chosen since the primary interest of this analysis was to investigate whether the previously uniformly reported higher levels of REM sleep during co-sleep in regularly co-sleeping couples as compared to individual sleep (13, 14, 33) could be confirmed in between subjects design. This led to the formulation of the primary hypothesis (being in a couple relationship is associated with increased REM sleep) for the testing of which only that exact effect direction (“greater”) is of interest. The secondary hypothesis (no effects on other sleep parameters) as well as the additional analyses (REM sleep fragmentation and number of sleep cycles) were tested using two-sided tests. Here the results of previous studies are more ambiguous (13, 14, 33).

There was only one primary hypothesis (impact of couple relationship on REM sleep). Thus, no adjustment for multiple testing was done.

All calculations were computed using “R” Version 3.6.1 (34). Propensity score matching was done using the “matchIT” package for “R” (35).

Significance levels were set at *p* < 0.05^*^, *p* < 0.01^**^, and *p* < 0.001^***^. Reported are means (±SD) in case of normally distributed data and median (IQR, lower to upper) in case of lacking normal distribution.

## Results

### Propensity Score Matching

**Figure 1:**
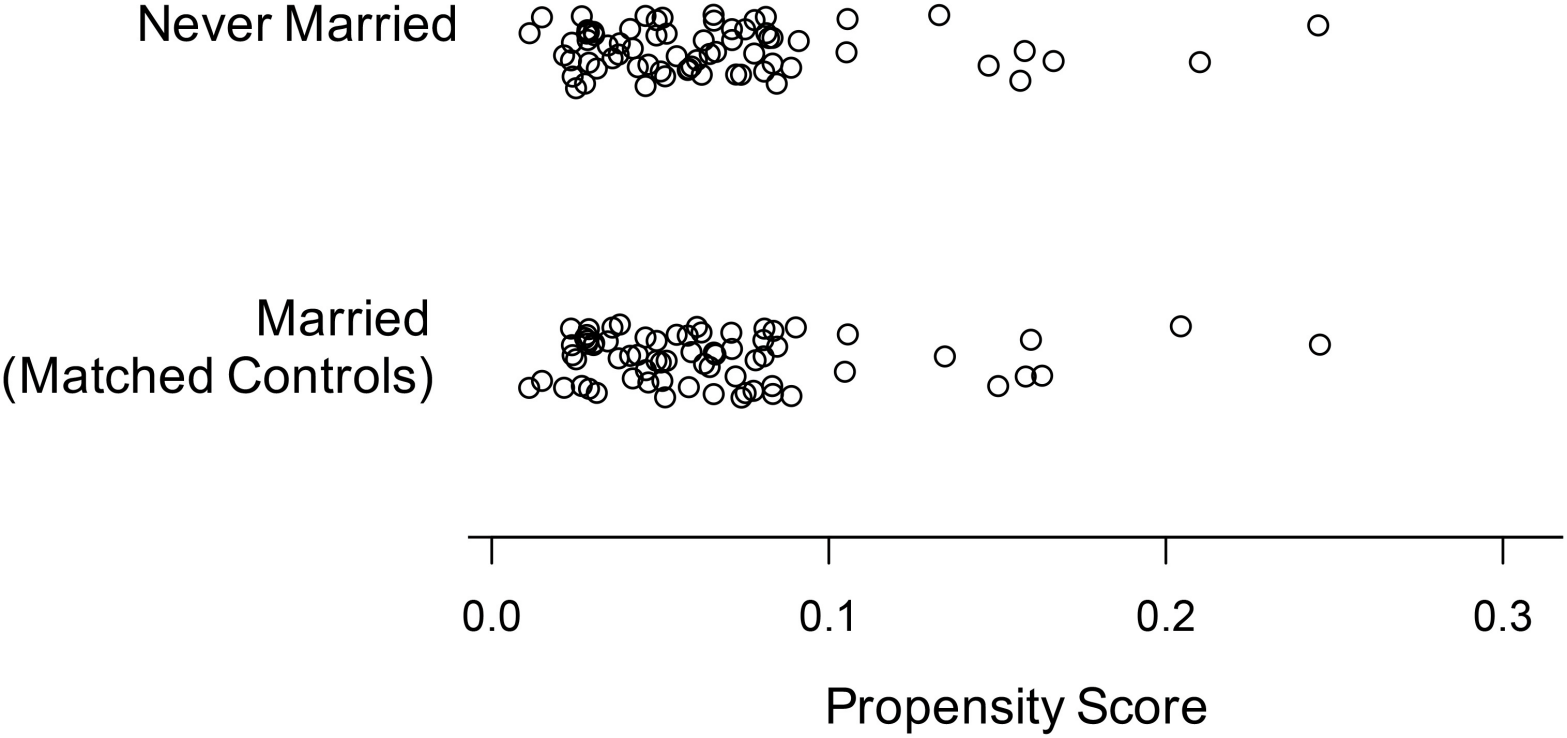
Propensity scores of the never married individuals and the matching married individuals after propensity score matching.

Propensity score matching resulted in two groups (*n* = 69 each) of married and never married individuals, respectively. The result of the matching procedure as represented by the propensity scores of the married and never married group is given in Figure 1. Moreover, the groups showed no significant differences regarding gender, ethnicity, age, education, subjective daytime sleepiness, Apnea-Hypopnea Index, use of antidepressants, and subjective health status as measured by the SF 36 sum score and all of its subscores (all *p*'s ≥ 0.27; see Table 1).

### REM Sleep and Other Polysomnographic Sleep Parameters

**Figure 2:**
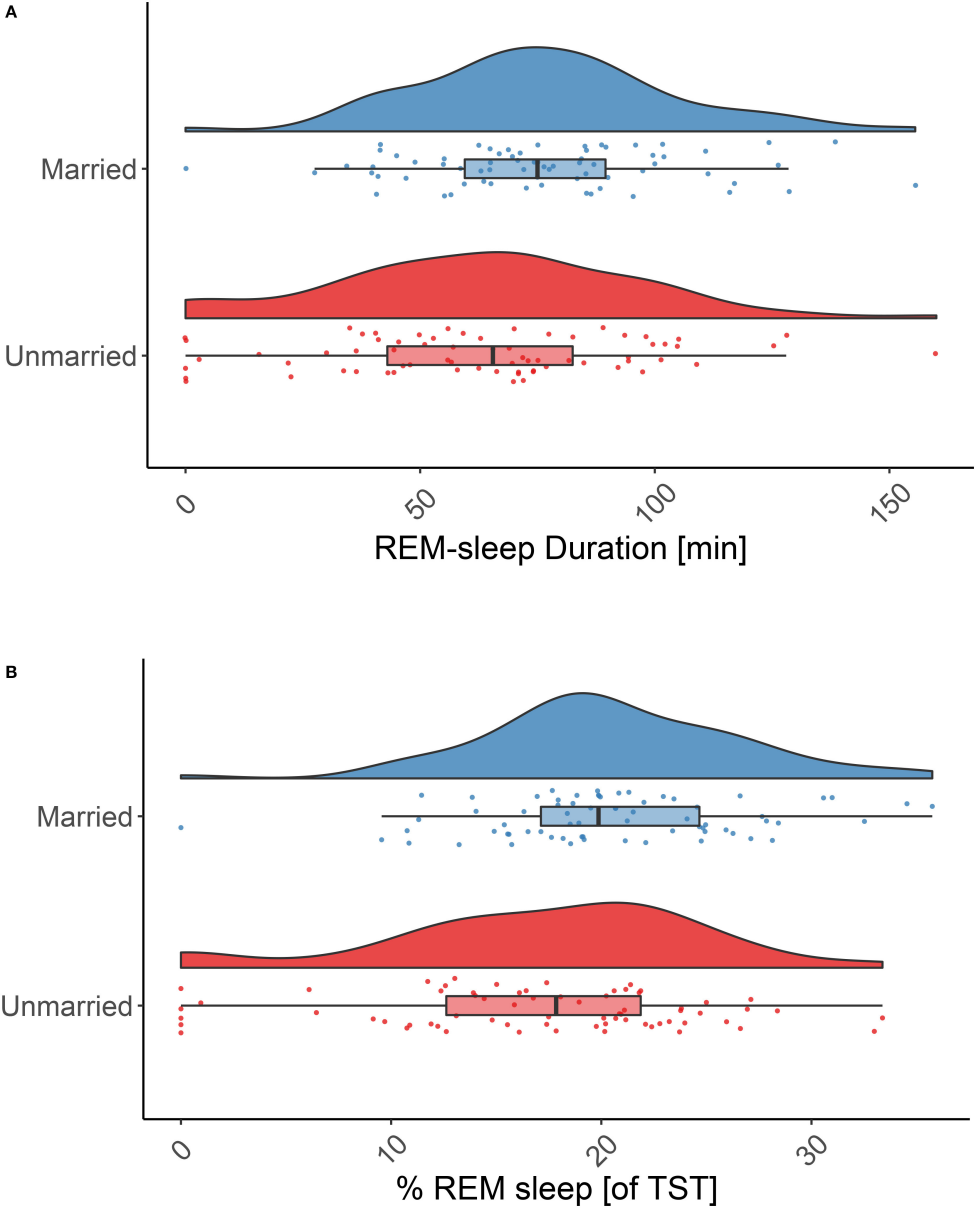
REM sleep duration in married and never married individuals. Absolute **(A)** and relative **(B)** REM sleep duration is significantly higher in married individuals (blue raincloud plots) than in never married individuals (red raincloud plots): 76.5 ± 27.7 min vs. 62.9 ± 33.1 min, *p* = 0.003; and 20.5 ± 6.2% vs. 17.1 ± 7.7% *p* = 0.001. Note: the effect remains significant if individuals with no or little REM sleep (≤3 min) are excluded (*p* = 0.023 absolute REM sleep amount and *p* = 0.015 relative amount of REM sleep). Tests: paired, one-sided *t*-tests.

Testing our main hypothesis, married individuals spent very significantly more time in REM sleep throughout the night as compared to never married individuals (76.5 ± 27.71 min vs. 62.8 ± 33.1 min, *p* = 0.003; Figure 2A). This also held true for the relative amount of REM sleep (20.5 ± 6.2% vs. 17.1 ± 7.7%, *p* = 0.001; Figure 2B). Since there was only one subject in the married group vs. six subjects in the never married group for which no or very little REM sleep (i.e., ≤3 min/night) was reported, we reanalyzed the data excluding these cases (and their matching partners). This led to a reduction of differences between the groups but the effect remained significant (*p* = 0.023 (absolute amount of REM sleep) and *p* = 0.015 (relative amount of REM sleep).

None of the other sleep-stage parameters was significantly altered (all *p'*s ≥0.29; Table 2).

**Table 2:** Polysomnographic sleep parameters.

	**Total sample (*n* = 138, *m* = 48)**	**Constantly married (*n* = 69, *m* = 25)**	**Never married (*n* = 69, *m* = 23)**	***p*-value (married vs. unmarried)**
		**[mean (± SD) or median (Q1–Q3)]**	**[mean (± SD) or median (Q1–Q3)]**	
Sleep onset Latency [min]	14.5 (8.5–25)	13.5 (9.3–24.8)	16 (8.5–24.7)	0.432
Total Sleep Time [min]	362 (± 60.8)	363 (± 51.7)	360 (± 68.6)	0.971
Sleep Efficiency [%]	85 (79.6–89.5)	85.2 (76.4–89.9)	85.9 (82.2–88.9)	0.29
Sleep Stage N1 [min]	15.8 (9.9–24.5)	16.0 (10.1–24.5)	15.5 (9.9–24.5)	0.931
Sleep Stage N2 [min]	208.6 (± 54.3)	210.9 (± 51.3)	206.3 (± 57.3)	0.575
Sleep Stage N3 [min]	64.5 (35.1–91.6)	64.0 (38.5–81.4)	64.8 (33.9–93.9)	0.85
Sleep Stage N1 [%]	4.3 (2.7–7)	4.3 (2.7–6.3)	4.3 (2.9–7)	0.872
Sleep Stage N2 [%]	57.0 (± 11.4)	56.9 (± 11.042)	57.1 (± 11.9)	0.755
Sleep Stage N3 [%]	17.0 (9.3–24.6)	17.0 (10.2–23.8)	17.8 (8.7–27.2)	0.694
REM Latency [min]	66.8 (48.6–88.3)	66.5 (51.0–80.5)	67.0 (46.5–107.0)	0.49
Wake after Sleep Onset [min]	44 (30.5–65.6)	43 (28.5–58)	46 (32–74)	0.29
Awakenings per hour	3.4 (2.6–4.3)	3.4 (2.8–4.1)	3.6 (2.6–4.4)	0.599

*No sleep stage other then REM sleep (see Figure 2) differs between married and never married individuals. Median (IQR, upper to lower) is given for parameters without normal distribution. Tests: two-sided, paired student t tests, or Wilcoxon signed-rank test*.

With respect to the additional analyses of number of REM periods (number of sleep cycles) and REM-sleep fragmentation, there was a significantly increased number of sleep cycles in married individuals [4 (4 to 5)] as compared to never married individuals [4 (3 to 5); *p* = 0.026] and the sleep cycles of married individuals were significantly shorter in duration [95.5 (84.1 to 112.0) min vs. 103.7 (87.6 to 133.2) min; *p* = 0.034]. This difference however became non-significant when the above-mentioned individuals with little or no REM sleep were excluded (all *p'*s ≥ 0.116).

Likewise, there was no significant difference in absolute or relative number of REM-sleep fragmentations between married and never married individuals (all *p'*s ≥ 0.433). The same held true when comparing the individual REM sleep periods between married and never married individuals with respect to absolute duration, and total and relative number of fragmentations (all *p*'s ≥ 0.090).

## Discussion

The present study reports that married individuals have significantly higher levels of absolute and relative REM sleep as compared to never married individuals. Secondly, no other sleep stage parameter differs significantly between the two groups. This means that both, the main as well the secondary hypothesis of the present analysis are confirmed. [Note that the observed increased REM-sleep duration remains in the non-pathological range (36) and is not associated with additional signs of REM-sleep disinhibition (e.g., reduced REM-sleep latency) which could indicate the presence of a mood disorder (37)].

These findings support the concept that being in a close couple relationship (which is associated with co-sleeping) is linked to increased REM sleep which has been put forward based on studies on regularly co-sleeping couples (13, 14, 33).

The present work complements the previous works and is an important step forward since the preceding studies used a within-subjects design that monitored the sleep of habitually co-sleeping couples in two sleeping arrangements: sleeping apart and sleeping together (13, 14, 33). This approach complicates the interpretation and generalizability of these studies since it is impossible to distinguish whether the observed higher levels in REM sleep when co-sleeping as compared to sleeping individually are due to a generalizable effect of sleeping in company or whether they are due to the specific alterations in the habitual sleeping environment (i.e., deprivation of a partner when sleeping individually). REM sleep is susceptible to psychosocial stress factors (6, 9, 11, 12). Changing the habitual sleeping arrangement by removing the partner could induce low-level stress that would lead to disruptions of REM sleep during individual sleep (13). Also, the artificial lab setting of the previous studies is an additional disruption of the habitual environment which might have interacted with the partner deprivation and might have amplified the stress effect. Additionally, the homogeneity of the samples regarding young age, health status, and ethnicity make the results of the previous studies less generalizable to the general population. These weaknesses have been discussed previously (13).

The present study overcomes these limitations:

1. It uses in-home polysomnography with no intervention to the usual sleeping arrangement so that participants are monitored in their usual sleeping arrangement and environment, excluding effects of partner deprivation and sleeping in a lab.
2. The sample is more heterogenous regarding age, ethnicity, and regarding health as compared to the previous studies and makes it more comparable to the general population. This increases generalizability.

This methodological approach of the present work excludes the possible stress-related explanation that limit the previous works. Thus, the here presented findings indicate a generalizable promoting effect of couple relationships on REM sleep. Thereby, the present work represents a necessary (but not sufficient) condition regarding the implications of the partner-related REM-sleep increase as proposed by Drews and colleagues (13, 33). These potential implications are, first, REM sleep acts as a mechanism through which close social relationships benefit mental health: Disrupted REM sleep has been argued to cause insomnia (2, 3), which in turn increases the risk for mental illnesses (e.g., mood disorders) (38). Second, a positive feedback loop between REM sleep and sociality: REM sleep has been reported to support emotional and episodic memory consolidation (39, 40), which [among other factors influenced by REM sleep (e.g., 41, 42)] are important for our ability to be social (43, 44). On the other hand, as exemplified by the previous studies on co-sleeping, sociality (such as sleeping with a partner) has been argued to impact REM sleep. Yet, given the above-mentioned limitations of the previous studies on habitually co-sleeping couples this has been a weak spot of the model. The present findings resolve that weakness.

An additionally relevant finding is the clarification of a partner-effect on other sleep stages. While a small pilot study has found effects on more sleep parameters including slow-wave sleep, sleep efficiency, and total sleep time (33), two larger studies report predominant effects on REM sleep (13, 14). The latter findings are supported by the present work.

However, there are ambiguities between the present work and these previous studies on co-sleeping couples that concern the microstructural correlates of the increase in total REM sleep when sleeping with a partner. Drews and colleagues (13) report decreased fragmentation of REM sleep, a non-significant decrease sleep stages N1–N3 (*p*-values between 0.26 and 0.5), and a non-significant (counterintuitive) increase in awakenings (*p* = 0.15). Monroe (14) on the other hand reports an increased number of REM periods (i.e., an increased number of sleep cycles), significantly reduced S4 sleep, no significant changes in S1–S3 sleep, and (likewise counterintuitively) significantly increased awakenings. The present work seemingly supports the increase in REM periods as microstructural correlate. However, exclusion of individuals with extremely low REM-sleep values (that might be caused by unknown confounding parameters) renders the results non-significant. Moreover, albeit not significant, the present work reports a decreased time of wake after sleep onset in married individuals and more similar values of non-REM sleep stages compared to the previous works. In sum, more research is needed to disentangle the microstructural correlates of REM increase in couple relationships and bed-sharing.

Besides these microstructural correlates of REM increase in close relationships, the present work also raises the question about the underlying mechanisms. Three mechanisms seem conceivable. First, a psychological mechanism: sleeping individually could represent a form of chronic stress impairing REM sleep and that is abrogated by a soothing effect of a partner. Second, a body-temperature-related mechanism: REM sleep is a state in which the body's capacity to maintain its temperature is impaired. A bed-partner might stabilize body temperature—as it has been hypothesized for the rock hyrax, which shows a similar increase of REM sleep when sleeping socially (45). Third, a circadian clocking mechanism: REM sleep is under strong circadian regulation (46). The circadian rhythm is known to be clocked by social cues (i.e., social zeitgebers) (47). Hence, the partner might impact circadian clocking which in turn might influence REM sleep.

Last but not least, our study suffers from limitations. First, the marital quality, work status, and menopausal status were not assessed, all of which have been reported to affect sleep (48–50). Second, the present work compares married individuals to never married individuals which means that the actual sleeping arrangement (bed-sharing vs. individual sleep) remains unknown. This could be seen as impairing comparability with the aforementioned studies on co-sleeping that directly manipulate the sleeping arrangement (13, 14, 33). Yet, it is of note that there is a high correlation between the marital statuses and sleeping arrangement: In the US ≥87% of the married individuals share a bed with their partner (18) (this number might be lower in elderly couples) and <5% of the elderly population (aged 50 and above, which is comparable to the present sample) are cohabiting (and co-sleeping) while not being married (17). Therefore, it seems rather unlikely that a significant number of neither never married but still bed-sharing individuals nor married but separately sleeping individuals confounds the results and interpretation of the present study.

Nevertheless, future studies should also include a group of couples that are habitually individually sleeping to better differentiate between the effects of being in a relationship (social setting) to sleep-setting-specific effects. A third important limitation of the present study is the cross-sectional design which precludes defining effect directions. While the previous interventional studies clearly indicate an effect of the sleeping arrangement on REM sleep (13, 14, 33) in the present study both directions are—theoretically—possible, i.e., REM sleep might increase the probability of getting married or vice versa. In fact, the hypothesized feedback loop of REM sleep and sociality would predict that both directions do occur.

Thus, future studies should use a long-term, longitudinal approach to actually retrace that postulated feedback loop.

In conclusion, the here presented analyses support and generalize the concept that couple relationships benefit REM sleep. The fact that this could be shown in a between-subjects design in comparison to (predominantly) habitually individually sleeping never married individuals complements previous studies and represents a necessary but not sufficient condition toward investigating a positive feedback loop of REM sleep and sociality as well as REM-sleep promotion as mechanism through which relationships benefit mental health—both of which should be addressed in future studies.

## Data Availability Statement

Publicly available datasets were analyzed in this study. This data can be found here: sleepdata.org.

## Ethics Statement

The present study is a re-analysis of a publicly available, anonymous dataset. Ethical review and approval was not required for the study on human participants in accordance with the local legislation and institutional requirements. Written informed consent for participation was not required for this study in accordance with the national legislation and the institutional requirements.

## Author Contributions

HD: conceptualization and writing—original draft preparation. HD and AD: design and methodology and statistical analysis and interpretation. AD: writing—review and editing and resources. All authors approved the submitted version.

## Conflict of Interest

The authors declare that the research was conducted in the absence of any commercial or financial relationships that could be construed as a potential conflict of interest.
